# Effect of exercise on vascular function in hypertension patients: A meta-analysis of randomized controlled trials

**DOI:** 10.3389/fcvm.2022.1013490

**Published:** 2022-12-21

**Authors:** Huayi Zhou, Shengya Wang, Changtao Zhao, Hui He

**Affiliations:** ^1^College of Sport and Human Science, Beijing Sport University, Beijing, China; ^2^Department of Physical Health and Arts Education, Ministry of Education, Beijing, China; ^3^China Institute of Sport and Health Science, Beijing Sport University, Beijing, China

**Keywords:** exercise, hypertension, vascular, meta-analysis, review

## Abstract

**Objective:**

The purpose of this study was to systematically evaluate the effect of exercise on vascular function in patients with pre- and hypertension.

**Methods:**

A systematic review of articles retrieved *via* the PubMed, Embase, EBSCO, and Web of Science databases was conducted. All the randomized controlled trials published between the establishment of the databases and October 2022 were included. Studies that evaluated the effects of exercise intervention on vascular function in patients with pre- and hypertension were selected.

**Results:**

A total of 717 subjects were included in 12 randomized controlled trials. The meta-analysis showed that in patients with pre- and hypertension, exercise can significantly reduce systolic blood pressure (SBP) (*MD* = –4.89; 95% CI, –7.05 to –2.73; *P* < 0.00001) and diastolic blood pressure (DBP) (*MD* = –3.74; 95% CI, –5.18 to –2.29; *P* < 0.00001) and can improve endothelium-dependent flow-mediated dilatation (*MD* = 2.14; 95% CI, 1.71–2.61; *P* < 0.00001), and exercise did not reduce pulse wave velocity (PWV) (*MD* = 0.03, 95% CI, –0.45–0.50; *P* = 0.92). Regression analysis showed that changes in exercise-related vascular function were independent of subject medication status, baseline SBP, age and duration of intervention.

**Conclusion:**

Aerobic, resistance, and high-intensity intermittent exercise all significantly improved SBP, DBP, and FMD in pre- and hypertensive patients, however, they were not effective in reducing PWV, and this effect was independent of the subject’s medication status, baseline SBP, age and duration of intervention.

**Systematic review registration:**

https://www.crd.york.ac.uk/PROSPERO/, identifier CRD42022302646.

## Introduction

As is well-known, hypertension, as a chronic disease, is one of the main risk factors for cardiovascular diseases. According to the World Health Organization (WHO), one billion people suffer from hypertension worldwide, and about nine million people currently die each year due to elevated blood pressure (1). The 2017 American College of Cardiology (ACC)/American Heart Association (AHA) blood pressure guidelines suggest that the cutoff point for hypertension diagnosis is systolic blood pressure (SBP) > 130 mm Hg or diastolic blood pressure (DBP) > 80 mm Hg (2). Available evidence suggests that cardiovascular prevalence is significantly higher in pre-hypertensive and hypertensive subjects than in healthy adults (3). Hypertension is highly linearly correlated with cardiovascular and cerebrovascular disease (CVD) and all-cause mortality (ACM), and blood pressure values have a linear relationship with the incidence of cardiovascular and CVDs; every 20 mm Hg increase in SBP or 10 mmHg increase in DBP doubled the risk of cardiovascular disease (4).

Elevated blood pressure can destroy vascular structure and function and the autocrine–paracrine relationship of the vascular wall (5). Hypertension is characterized by endothelial dysfunction and arterial remodeling, which lead to increased vascular wall thickness and arterial stiffness, which in turn increase blood pressure, forming a vicious cycle (6–9). SBP, DBP, FMD [Flow-mediated dilation (FMD)], and PWV (Pulse wave velocity) are correlated with the incidence of hypertension (2, 4, 5, 10–13). Vascular endothelium plays an important role in regulating angiogenesis, inflammatory response, angiogenesis, and peripheral vascular resistance (14). Endothelium-dependent FMD is one of the indicators of endothelial function and an increase in FMD is associated with improved cardiovascular disease, with studies showing that a 1% increase in FMD is associated with a 13% reduction in cardiovascular risk (15). High blood pressure will disorder vascular endothelial cells and consequently lead to decreased FMD (8). Arterial stiffness is a risk factor for hypertension. PWV refers to the conduction velocity of the pressure wave propagating along the wall of the aorta with each pulse ejection, and it is a non-invasive index for evaluating the stiffness of arterial vessels, and PWV measurements are considered more sensitive than conventional blood pressure measurements (16). Theoretically, increased arterial stiffness is related to the loss of arterial elasticity and decreased compliance, and increased blood pressure will lead to vascular wall remodeling and vascular dysfunction, in order to compensate for changes in vascular wall stress, thus further aggravating arterial stiffness (17). Therefore, improving vascular function is essential for management and prevention strategies in patients with pre-hypertension and hypertension.

A large number of experimental and observational studies have demonstrated that lifestyle-based interventions, such as increased physical activity and dietary approaches, are effective in preventing CVD (18–20). Exercise, as a non-drug treatment, can effectively decrease hypertension. Studies have shown that regular exercise can improve cardiovascular health and reduce blood pressure; among many forms of exercise, aerobic exercise (AE) has become the primary recommendation for the prevention and treatment of hypertension (21–23). The lifestyle-management guidelines published by the ACC and AHA suggest that patients with cardiovascular diseases such as hypertension should perform 40 min of moderate-intensity AE three to four times per week for at least 12 weeks (24). Studies have demonstrated that regular AE can reduce the impairment of endothelium-dependent vasodilation (25) and atherosclerosis (26) in patients with hypertension. Unlike the research on AE, the research on resistance training (RT) is controversial. The AHA (27), American College of Sports Medicine (ACSM) (28), European Society of Hypertension (ESH/ESC) (29), and Canadian Hypertensive Education Program (CHEP) (30) recommend RT only as a supplement to AE in adults with hypertension. Other studies have shown that RT reduces blood pressure in adult hypertensive patients and that this reduction may be similar to that associated with AE (31, 32). Although RT can effectively reduce blood pressure, its ability to improve vascular endothelial function and vascular stiffness in patients with hypertension is debated. Two recent studies have shown that high-intensity resistance exercise reduces FMD and increases arterial stiffness (33, 34), while others have demonstrated that resistance exercise does not affect the increase or decrease in PWV (35). Few studies have investigated the effect of high-intensity interval training (HIIT) on vascular function in hypertensive patients. Some studies have shown that, compared with AE, HIIT can more effectively improve blood pressure, endothelial function (36) and arterial stiffness (37). Although research has shown that exercise reduces hypertension, few studies have focused on the effects of exercise on vascular function in patients with hypertension, and it is not clear which mode of exercise (AE, RT, or HIIT) has the best effect on vascular function in such patients. Therefore, in this study, we investigated the different effects of exercise on endothelial function and arterial stiffness in pre- and hypertensive patients based on the fact that exercise can lower blood pressure and to provide most suitable exercise advice for preventing vascular pathology in patients with hypertension.

## Methods

The writing of this article strictly followed the Preferred Reporting Items for Systematic Reviews and Meta-analyses (PRISMA) guidelines (38).

### Data sources and searches

In this study, two experienced researchers conducted a literature search in PubMed, Embase, EBSCO, Web of Science, and other databases. The retrieval time was from the establishment of the database to October 2022, and the references included in the literature were tracked. The search terms were as follows: exercises OR physical activity OR isometric exercises OR AE OR exercise training AND vascular endothelium OR capillary endothelium OR endothelium, capillary AND vascular OR blood vessels OR vessel, blood OR vessels, blood.

### Study selection

Studies were eligible for inclusion if they met all the following criteria: (1) experimental type: RCT, (2) subjects: patients with hypertension or prehypertension, (3) intervention: the experimental group exercised and the control group did not exercise and the exercise duration of the experimental group was more than 4 weeks, and (4) outcome indicators: SBP, DBP, FMD, PWV. Studies were excluded if (1) they lacked an RCT, (2) they lacked a blank control group, (3) repeated studies, (4) they provided no outcome indicators, (5) accompanied by myocardial infarction, chronic heart failure, arrhythmia and other cardiovascular diseases, (6) they were reviews or conference reports, (7) they contained animal experiments, or (8) they were written in a language other than English.

### Data screening and extraction

According to the retrieval strategy, all the retrieved studies were imported into the literature-management software EndNote and duplicate studies were deleted. Two of the researchers screened the literature according to the inclusion and exclusion criteria. The determination to exclude a study was made by reading the title and abstract, downloading the full text of the study, and reading the full text in detail. The two researchers compared their screening results. If the screening results were inconsistent, a third researcher was enlisted to discuss the decision. In case of incomplete data or unclear description of the finally included literature, the author of the literature shall be contacted by e-mail. The outcome indicators were extracted from the studies that were ultimately included, and the tables were designed and completed by the two researchers independently. The extracted content consisted of (1) basic information about the included studies: first author, publication date, etc., (2) the subjects’ baseline characteristics: age, sex, medication status and blood pressure, (3) the intervention measures applied to the experimental group: exercise duration, exercise mode, and exercise intensity, and (4) the relevant outcome indicators and outcome data.

### Quality assessment

Using the Cochrane Collaboration’s tool for assessing the risk of bias (39), the included studies were evaluated for seven aspects of methodological quality, as follows: (1) random sequence generation, (2) allocation hidden, (3) blind method for subjects and experimentalists, (4) blinding of outcome assessment, (5) incomplete data report, (6) the results of selective reporting, and (7) other sources of bias.

### Statistical analysis

In this study, the included literature data were uniformly converted to mean ± standard deviation (M ± SD). RevMan 5.3 and STATA 12.0 software provided by the Cochrane Library Collaboration network (StataCorp., College Station, Texas, USA) the difference of mean ± standard deviation (M ± SD) before and after intervention was analyzed. The heterogeneity of the included data was assessed by calculating the value of *I*^2^. The Cochran Handbook suggests that if *I*^2^ < 50% and *Q*-test *P* > 0.1, the heterogeneity among different study groups is small; if this was the case, a fixed effects model was adopted for analysis. If *I*^2^ ≥ 50% or *Q*-test *P* ≤ 0.1, both of which indicate a high level of heterogeneity among the study groups, a random effects model was adopted. SBP, DBP, FMD, and PWV outcomes were continuous variables, and the measurement units were the same. Therefore, a mean difference (MD) effect scale and a 95% confidence interval (CI) were used for the statistics. Subgroup analysis and sensitivity analysis were carried out according to the results of possible sources of heterogeneity, and a publication-bias test was carried out. Egger’s method was used for quantitative testing. If *P* < 0.05, there was publication bias, and this bias was eliminated using the cut-and-complement method.

## Results

### Literature search and selection

**FIGURE 1:**
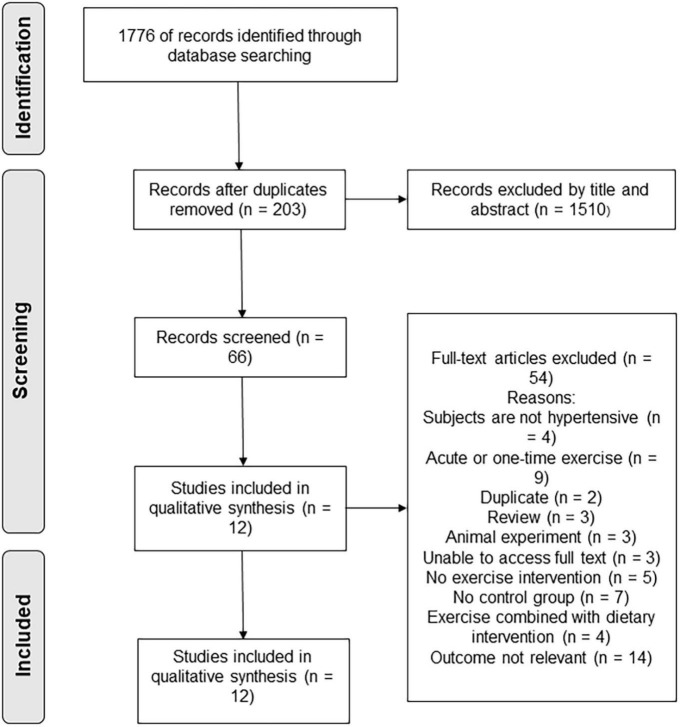
Flowchart of the literature-retrieval protocol.

According to the established literature retrieval strategy, a total of 1,779 articles were retrieved. The databases used and the number of articles detected by each database are as follows: PubMed (*n* = 594), EMBASE (*n* = 259), Web of Science (*n* = 827), EBSCO (*n* = 99). The literature-management software EndNote was used to eliminate 203 duplicate articles, and 1,510 articles were deleted after reading their titles and abstracts. After the full text of the articles was read, 54 articles were removed and 12 were ultimately included, as shown in Figure 1.

### Description of the included trials

Based on the 12 included studies, a total of 717 subjects (469 in the exercise group and 248 in the control group) were included. Among these studies, one article included postmenopausal women (40, 41), one included type 2 diabetes mellitus (T2DM) (42), and two included metabolic syndrome (12, 43). Four of the studies used two exercise programs (37, 42–44), two used three exercise programs (12, 40), four used an RT intervention (10, 12, 41, 44), 8 used an AE intervention (37, 40, 42–47), and five used a HIIT intervention (11, 12, 37, 42, 43). The members of the control group did not perform an exercise intervention and maintained their previous lifestyle. One intervention lasted 6 weeks (41), one lasted 8 weeks (44), four lasted 12 weeks (42, 45–47), two lasted 16 weeks (37, 43), and two lasted 24 weeks (11, 40). Nine studies reported an exercise frequency of 3 days/week (10–12, 37, 42–46), one reported an exercise frequency of 3–4 days/week (40), one reported an exercise frequency of 5 days/week (46), and one reported an exercise frequency of 6 days/week (41). There were 7 studies in which subjects took anti-hypertensive medicines, and 5 studies in which subjects did not take anti-hypertensive medicines (Table 1).

**TABLE 1:** Characteristics of the included trials.

			Control		Exercise				
Study	Patients	Medication status	Sample size (M/F)	Age	Sample size (M/F)	Age	Intervening measure and intensity	Intervention duration	Outcome indicator
Afousi et al. (42)	T2DM, SBP: 120–159 mm Hg, DBP: 80–99 mm Hg	aHTN	17 (8/9)	54.24 ± 5.61	LVHIIT: 18 (9/9)	54.78 ± 6.19	LVHIIT, 85–90% HR_*max*_, 2 min at 55–60% HR_*max*_, 1.5 min, 12 intervals	3 d/w, 12 w	SBP, DBP, FMD
					CMIT: 17 (7/10)	53.12 ± 4.84	AE (CMIT), 40% HR_*max*_, 42 min		
Craighead et al. (41)	SBP: 120–139 mm Hg, DBP: 80–89 mm Hg	aHTN	18 (10/8)	67 ± 2	18 (9/9)	67 ± 2	Week 1: 55% PIMAX, week 2: 65% PIMAX, week 3: 67.5% PIMAX	6 d/w, 6 w	SBP, DBP, FMD, PWV
Glodzik et al. (45)	SBP: 131.65 ± 3.57 mm Hg, DBP: 80.65 ± 5.21 mm Hg	None	14 (8/6)	45.0 ± 3.41	31 (23/8)	44.3 ± 5.57	AE, 40–65% HRR	3 d/w, 12 w	SBP, DBP, FMD
Liang et al. (46)	SBP: 140–159 mm Hg, DBP: 90–99mm Hg	None	66 (66/0)	18–40	75 (75/0)	18–40	AE, 75% maximal metabolic equivalent	5 d/w, 12 w	SBP, DBP, FMD, PWV
Westhoff et al. (47)	SBP ≥ 140 mm Hg	aHTN	12 (5/7)	68.4 ± 9.7	12 (6/6)	66.1 ± 4.0	AE (aerobic arm cycling), target lactate concentrations of 2.0∼0.5 mmol/L	3 d/w, 12 w	SBP, DBP, FMD
Guimarães et al. (37)	SBP: 120–139 mm Hg, DBP:80–89 mm Hg	aHTN	11 (9/2)	47 ± 6	Continuous: 16 (9/7)	50 ± 8	AE, 60% HRR	40 min/d, 3 d/w, 16 w	SBP, DBP, PWV
					Interval: 16 (12/4)	45 ± 9	HIIT, 50% (2 min) and 80% (1 min) HRR		
Beck (44)	SBP: 120–139 mm Hg, DBP:80–89 mm Hg	None	15 (10/5)	21.6 ± 2.9	PHET: 13 (9/4)	20.1 ± 1.1	PHET, AE, 65 –85% HR_max_	60 min/d, 3 d/w, 8 w	SBP, DBP, FMD
					PHRT: 15 (11/4)	21.1 ± 2.5	PHRT, 8–12 RM		
Swift et al. (40)	Postmenopausal, SBP: 120–159 mm Hg, overweight	None	23 (0/23)	56.8 ± 5.4	4 kcal/kg/week: 68 (0/68)	57.4 ± 5.8	AE, 50% VO_2_ peak	3 or 4 d/w, 24 w	SBP, DBP, FMD
					8 kcal/kg/week: 32 (0/32)	55.9 ± 6.0			
					12 kcal/kg/week: 32 (0/32)	56.3 ± 6.8			
Cahu Rodrigues et al. (10)	SBP: 120–139 mm Hg, DBP:80–89 mm Hg	aHTN	17	59 ± 2	16	61 ± 2	RT (isometric handgrip training), 30% of maximal voluntary contraction	3 d/w, 12 w	SBP, DBP, PWV
Mora-Rodriguez et al. (11)	SBP ≥ 130 mm Hg and/or DBP ≥ 85 mm Hg	None	23	53.5 ± 8.9	23	53.5 ± 8.9	HIIT, 4 min 90% HR_max_, 3-min recovery at 70% HRmax, 4 intervals	45 min/d, 3 d/w, 24 w	SBP, DBP, PWV
Stensvold et al. (12)	SBP ≥ 130 mm Hg and/or DBP ≥ 85 mm Hg, syndrome	OHG, aHTN	11	47.3 ± 10.2	AIT:11	49.9 ± 10.1	AIT, 4 min at 90–95% of HR_peak_, 3-min recovery at 70% of HR_peak_, 4 intervals		SBP, DBP, FMD
					RT:11	50.9 ± 7.6	RT, 80% 1-RM, 8–12 repetitions, 3 sets	3 d/w, 12 w	
					COM:10	52.9 ± 0.4	COM, AIT twice a week and RT once a week		
Tjønna et al. (43)	SBP ≥ 130 mm Hg and/or DBP ≥ 85 mm Hg, syndrome	OHG, aHTN	9 (5/4)	49.6 ± 9.0	CMIT:8 (4/4) AIT:11 (4/7)	52.0 ± 10.6 55.3 ± 13.2	CMIT, 70% HR_max_ AIT, 4-min 90% HR_max_, 3-min 70% of HR_max,_ 4 intervals	3 d/w, 16w	SBP, DBP, FMD

aHTN, antihypertensive drugs; OHG, oral hypoglycemic agents; T2DM, Type 2 Diabetes Mellitus; M, male; F, female; HIIT, High Intensity Interval Training; CMIT, continuous moderate intensity training; PHET, endurance exercise training; PHRT, resistance exercise training; AIT, aerobic interval training; RT, resistant training; COM, combination of AIT and RT; AE, aerobic exercise; HRR, heart rate reserve; HRmax, maximal heart rate; HRpeak, peak heart rate; VO_2_ peak, Peak oxygen uptake; PIMAX, maximal inspiratory pressure; RM, repetition maximum.

### Methodological quality assessment

**FIGURE 2:**
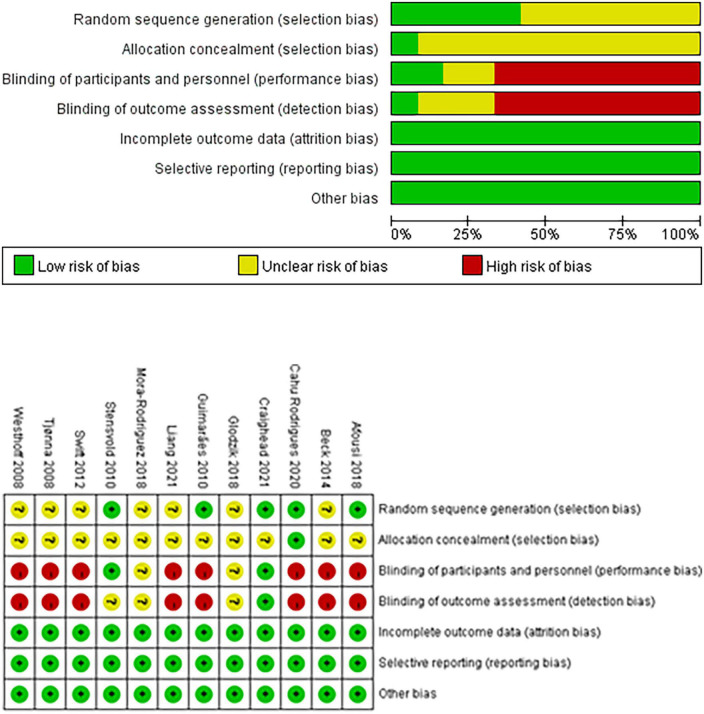
Cochrane risk-of-bias evaluation chart.

The included articles were evaluated for risk bias. Five of the 12 included studies mentioned the use of random grouping and involved random sequence generation. Allocation hiding was achieved in one study, whereas random sequence generation was achieved in the other studies. Two studies involved blinding of participants and personnel, whereas the other studies failed to achieve blinding of participants and personnel. Blinding of outcome assessment was performed in one study but not in the others. Incomplete outcome data attrition bias was achieved in 12 studies. Selective reporting was used in 12 studies. Twelve studies determined that no other bias existed (Figure 2).

### Synthesis of the results

#### Analysis of systolic blood pressure

**FIGURE 3:**
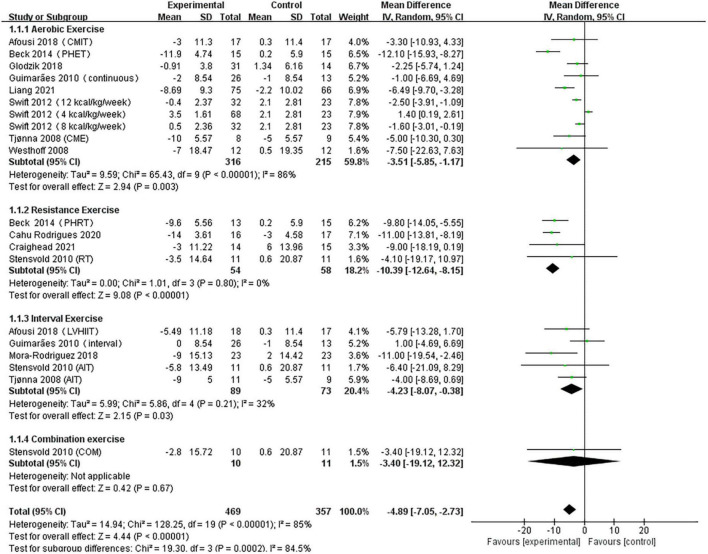
Forest plot of postintervention SBP-value comparison between exercise and control groups. SD, standard deviation; Std, standardized; IV, inverse variance; CI, confidence interval; CME, continuous moderate exercise; LVHIIT, low-volume high-intensity interval training.

A pooled analysis of the 12 articles (*n* = 717 participants, 469 in the exercise group and 248 in the control group) that assessed the effects of exercise on SBP was performed. We used random effects models for pooled effect estimates. The combined effect size MD of –4.89 (95% CI, –7.05 to –2.73; *P* < 0.00001; *I*^2^ = 85%; *P* for heterogeneity < 0.00001) indicated that the MD of SBP was statistically significant and that, compared with the control group, exercise could significantly decrease the SBP of pre- and hypertensive patients. The subgroup analysis showed that 10 studies (*n* = 531) used AE with a combined effect MD of –3.51 (95% CI, –5.85 to –1.17; *P* = 0.003; *I*^2^ = 86%; *P* for heterogeneity < 0.00001), suggesting that AE can significantly decrease SBP in pre- and hypertensive patients. Four studies (*n* = 112) used RT with a combined effect MD of –10.39 (95% CI, –12.64 to –8.15; *P* < 0.00001; *I*^2^ = 0%; *P* for heterogeneity = 0.80), suggesting that RT can significantly decrease SBP in pre- and hypertensive patients. Five studies (*n* = 162) used HIIT with a combined effect MD of –4.23 (95% CI, –8.07 to –0.38; *P* = 0.007; *I*^2^ = 32%; *P* for heterogeneity = 0.21), suggesting that HIIT can significantly decrease SBP in pre- and hypertensive patients. One study (*n* = 21) used combination exercise with a combined effect MD of –3.40 (95% CI, –19.12 to 12.32; *P* = 0.67), suggesting that there was no statistically significant difference in SBP between the two groups (Figure 3).

#### Analysis of diastolic blood pressure

A pooled analysis of the 12 articles (*n* = 717 participants, 469 in the exercise group and 248 in the control group) that assessed the effects of exercise on DBP was performed. We used random effects models for pooled effect estimates. The combined effect size MD of –3.74 mmHg (95% CI, –5.18 mmHg to –2.29 mmHg; *P* < 0.00001; *I*^2^ = 87%; *P* for heterogeneity < 0.00001) indicated that the MD of DBP was statistically significant and that, compared with the control group, exercise could significantly improve the DBP of pre- and hypertensive patients. The subgroup analysis showed that 10 studies (*n* = 531) used AE with a combined effect MD of –2.77 mmHg (95% CI, –4.22 mm Hg to –1.32 mm Hg; *P* = 0.0002; *I*^2^ = 83%; *P* for heterogeneity < 0.00001), suggesting that AE can significantly decrease DBP in pre- and hypertensive patients. Four studies (*n* = 112) used RT with a combined effect MD.

**FIGURE 4:**
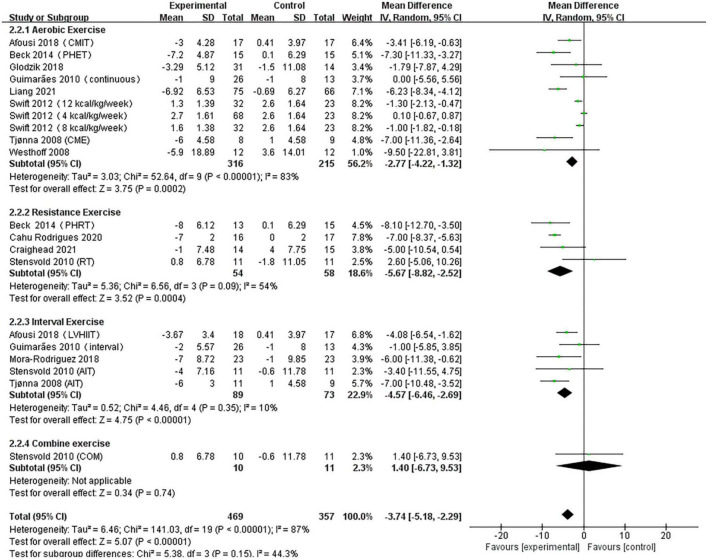
Forest plot of postintervention DBP-value comparison between exercise and control groups. SD, standard deviation; Std, standardized; IV, inverse variance; CI, confidence interval.

Of –5.67 mm Hg (95% CI, –8.82 to –2.52 mm Hg; *P* = 0.0004; *I*^2^ = 54%; *P* for heterogeneity = 0.09), suggesting that RT can significantly decrease SBP in pre- and hypertensive patients. Five studies (*n* = 162) used HIIT with a combined effect MD of –4.57 mm Hg (95% CI, –6.46 to –2.69 mm Hg; *P* < 0.00001; *I*^2^ = 10%; *P* for heterogeneity = 0.35), indicating that HIIT can significantly decrease DBP in pre- and hypertensive patients. One study (*n* = 21) used combination exercise with a combined effect MD of 1.40 mm Hg (95% CI, –6.73 to 9.53 mm Hg; *P* = 0.74), suggesting that there was no statistically significant difference in DBP between the two groups (Figure 4).

#### Analysis of flow-mediated dilation

**FIGURE 5:**
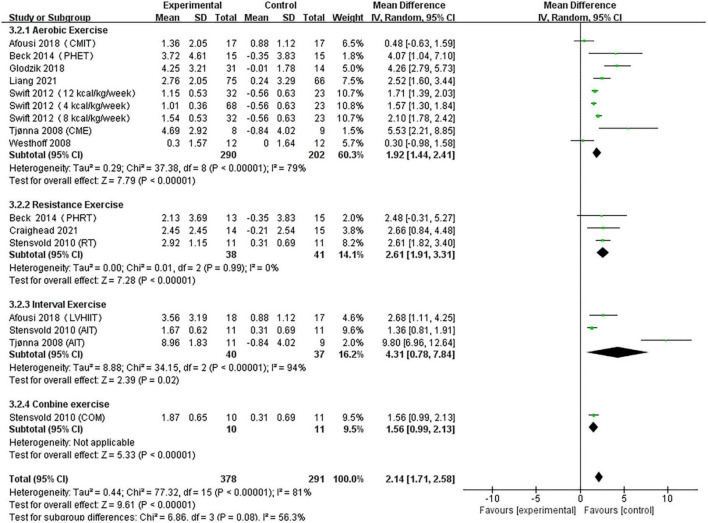
Forest plot of postintervention FMD-value comparison between exercise and control groups. SD, standard deviation; Std, standardized; IV, inverse variance; CI, confidence interval.

A pooled analysis of the nine articles (*n* = 571 participants, 378 in the exercise group and 193 in the control group) that assessed the effects of exercise on FMD was performed. We used random effects models for pooled effect estimates. The combined effect size MD was 2.14 (95% CI, 1.71–2.61; *P* < 0.0001; *I*^2^ = 81%; *P* for heterogeneity < 0.00001), indicating that the MD of FMD was statistically significant and that, compared with the control group, exercise could significantly improve the FMD of patients. Subgroup analysis showed that seven studies (*n* = 446) used AE, with a combined effect MD of 1.92 (95% CI, 1.44–2.41; *P* < 0.00001; *I*^2^ = 79%; *P* for heterogeneity < 0.00001), suggesting that AE can significantly improve FMD in patients. Three studies (*n* = 79) used RT, with a combined effect MD of 2.61 (95% CI, 1.91–3.31; *P* < 0.00001; *I*^2^ = 0%; *P* for heterogeneity = 0.99), suggesting that RT can significantly improve FMD in patients. Three studies (*n* = 77) used HIIT with a combined effect MD of 4.31 (95% CI, 0.78–7.84; *P* = 0.02; *I*^2^ = 94%; *P* for heterogeneity < 0.00001), indicating that HIIT can significantly improve FMD in patients. One study (*n* = 21) used combination exercise with a combined effect MD of 1.56 (95% CI, 0.99–2.13; *P* < 0.00001), indicating that combination exercise can significantly improve FMD in patients (Figure 5).

#### Analysis of pulse wave velocity

**FIGURE 6:**
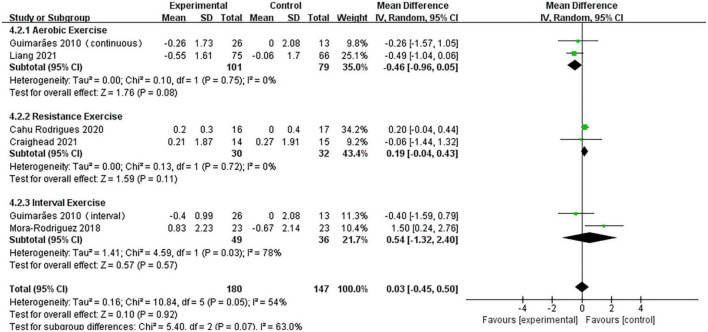
Forest plot of postintervention PWV-value comparison between exercise and control groups. SD, standard deviation; Std, standardized; IV, inverse variance; CI, confidence interval.

A pooled analysis of the five articles (*n* = 314 participants, 180 in the exercise group and 134 in the control group) that assessed the effects of exercise on PWV was performed. We used random effects models for pooled effect estimates. The combined effect size MD of 0.03 (95% CI, –0.45 to 0.50; *P* = 0.92; *I*^2^ = 54%; *P* for heterogeneity = 0.05) indicated that the MD of PWV was not statistically significant. The subgroup analysis showed that two studies (*n* = 180) used AE, with a combined effect MD of –0.46 (95% CI, –0.96 to 0.05; *P* = 0.08; *I*^2^ = 0%; *P* for heterogeneity = 0.75), suggesting that there was no statistically significant difference in PWV between the two groups and that AE cannot significantly improve PWV in hypertensive patients. Two studies (*n* = 62) used RT with a combined effect MD of 0.19 (95% CI, –0.04 to 0.43; *P* = 0.11; *I*^2^ = 0%; *P* for heterogeneity = 0.72), indicating that there was no statistically significant difference in PWV between the two groups. Two studies (*n* = 85) used HIIT, with a combined effect MD of 0.54 (95% CI, –1.32 to 2.40; *P* = 0.57, *I*^2^ = 78%; *P* for heterogeneity = 0.03), indicating that there was no statistically significant difference in PWV between the two groups (Figure 6).

### Meta-regression analysis

Univariate meta-regression analysis showed that changes in FMD and PWV associated with exercise were independent of the subjects’ medication status, basal SBP, age and duration of exercise (Table 2). Further subgroup analysis of subjects included in the study showed that exercise significantly improved FMD in hypertensive patients taking (*MD* = 2.36, *I*^2^ = 81%, *P* < 0.00001) or not taking (*MD* = 2.11, *I*^2^ = 73%, *P* < 0.00001) antihypertensives, with a basal SBP ≥ 140 mm Hg (*MD* = 1.94, *I*^2^ = 55%, *P* < 0.00001) or SBP < 140 mm Hg (*MD* = 2.34, *I*^2^ = 85%, *P* < 0.00001), and aged ≥ 50 (*MD* = 2.01, *I*^2^ = 82%, *P* < 0.00001) or < 50 (*MD* = 2.81, *I*^2^ = 80%, *P* < 0.00001) years, with similar effects. In contrast, there was no significant change in PWV after the exercise intervention in hypertensive subjects taking (*MD* = –0.17, *I*^2^ = 88% *P* = 0.08) or not taking (*MD* = 2.36, *I*^2^ = 81%, *P* < 0.00001) antihypertensives, with basal SBP ≥ 140 mm Hg (*MD* = –0.49, *P* = 0.08) or SBP < 140 mm Hg (*MD* = 0.20, *I*^2^ = 29%, *P* = 0.42), aged ≥ 50 (*MD* = 0.27, *I*^2^ = 42%, *P* = 0.38) or < 50 (*MD* = –0.46, *I*^2^ = 0%, *P* = 0.08) years, and with exercise duration ≥ 12 weeks (*MD* = 0.04, *I*^2^ = 63%, *P* = 0.89) or < 12 weeks (*MD* = –0.06, *P* = 0.93) (Table 3).

**TABLE 2:** Meta-regression analyses on FMD and PWV in studies included in the meta-analysis.

Variables	FMD			PWV		
		
	N	T	P	N	T	*P*
Medication	9	0.79	0.45	4	0.24	0.82
Baseline SBP ≥ 140 mm Hg	5	2.10	0.06	1	–0.23	0.84
Age ≥ 50	12	0.49	0.63	4	0.77	0.52
Exercise duration ≥ 12 week	3	−0.24	0.81	1	0.58	0.62

**TABLE 3:** Subgroup analyses for FMD and PWV in studies included in the meta-analysis.

	FMD				PWV			
		
Group	*N*	*MD*	*I* ^2^	*P* overall change	*N*	*MD*	*I* ^2^	*P* overall change
Medication	16	2.14	81%	*P* < 0.00001	6	0.03	54%	*P* = 0.92
Yes	9	2.36	85%	*P* < 0.00001	4	0.16	0%	*P* = 0.18
No	7	2.11	73%	*P* < 0.00001	2	–0.17	88%	*P* = 0.50
Baseline SBP	16	2.14	81%	*P* < 0.00001	6	0.03	54%	*P* = 0.92
≥140 mm Hg	5	1.94	55%	*P* < 0.00001	1	–0.49	—	*P* = 0.08
<140 mm Hg	11	2.34	85%	*P*<0.00001	5	0.20	29%	*P* = 0.42
Age	16	2.14	81%	*P*<0.00001	6	0.03	54%	*P* = 0.92
≥50 years	12	2.01	82%	*P* < 0.00001	4	0.27	42%	*P* = 0.38
<50 years	4	2.81	80%	*P*<0.00001	2	–0.46	0%	*P* = 0.08
Exercise duration	16	2.14	81%	*P* < 0.00001	6	0.03	54%	*P* = 0.92
≥12 weeks	3	2.06	80%	*P* < 0.00001	2	0.04	63%	*P* = 0.89
<12 weeks	13	2.76	89%	*P* = 0.07	1	–0.06	—	*P* = 0.93

### Sensitivity analysis

To test the stability and reliability of the meta-analysis results, the sensitivity of exercise to SBP, DBP, FMD, and PWV was analyzed. After the removal of each study, the results of the sensitivity analysis showed that the results were relatively robust, as shown in Supplementary Figure 1.

### Evolution of publication bias

Stata version 12.0 was used for the publication bias test, a funnel diagram was made for SBP, DBP, FMD and PWV, and Egger’s method was used for the quantitative test. The funnel diagram of exercise on SBP was asymmetric. Egger’s test *t* = −2.35, *P* > 0.030, and there is publication bias test. We used the trim and fill method to eliminate the publication bias of exercise on SBP. After the trim and fill method was performed, the publication bias was eliminated, the combined effect amount did not change, and there was still a significant difference (*MD* = −4.890; 95% CI, −7.047 to −2.733; *P* = 0.000). The funnel diagram of exercise on DBP was asymmetric. Egger’s test *t* = −2.33, *P* > 0.031, and there is publication bias test. We used the trim and fill method to eliminate the publication bias of exercise on SBP. After the trim and fill method was performed, the publication bias was eliminated, the combined effect amount did not change, and there was still a significant difference (*MD* = −3.852, 95% CI, −5.297 to −2.406; *P* = 0.000). The funnel diagram of exercise on FMD was asically symmetrical, Egger’s-test *P* was 0.083, and no publication bias was found. The funnel diagram of exercise on PWV was basically symmetrical, Egger’s-test *P* was 0.770, and no publication bias was found, as shown in Supplementary Figure 2.

## Discussion

This review summarizes the effects of different exercise modes on vascular function in pre- and hypertensive patients by incorporating relevant RCT studies. The results of this study showed that (1) AE, RT, and HIIT significantly increased FMD and decreased blood pressure in pre- and hypertensive patients; (2) AE, RT, and HIIT were not effective in reducing PWV in pre- and hypertensive patients.

### Blood pressure

It is well-known that exercise can reduce blood pressure. Its physiological mechanism is that under the neurohumoral regulation, the activity of sympathetic nerve decreases and the diameter of arterial lumen increases, so as to reduce the resistance of peripheral blood vessels and blood pressure (48). In the current study, AE was the most highly recommended exercise for preventing and improving blood pressure (23). Studies have shown that AE in patients with hypertension can reduce SBP and DBP by about 3.5 and 3 mm Hg, respectively (49). Our study shows that AE significantly reduces blood pressure (SBP –3.51 mm Hg and DBP –2.77 mm Hg) in patients with hypertension, which is similar to the results of previous studies. Early meta-analyses showed that AE reduces SBP and DBP in hypertensive patients by reducing vascular resistance and inhibiting the sympathetic nervous system and the renin-angiotensin system (50). The ability of RT to decrease hypertension is debated. Studies have shown that RT can reduce peripheral vascular resistance, thus reducing systemic blood pressure (6, 51). However, some meta-analyses have shown that although RT can reduce blood pressure, it is not as effective as AE (52). Interestingly, our results show that RT is more effective than AE in reducing blood pressure (SBP and DBP were –10.39 and –4.57 mm Hg, respectively) in hypertensive patients, which is quite different from the results of previous studies. However, some studies have shown that RT can reduce blood pressure in hypertensive patients, and the reduction may be similar to that associated with AE (31, 32, 53–56). A recent meta-analysis showed that RT can significantly reduce blood pressure, especially diastolic blood pressure (57). In these four studies, Cahu Rodrigues (10) used isometric handgrip training (IHT) three times a week for 12 weeks, which significantly reduced blood pressure in hypertensive patients (SBP and DBP were –14 and –7 mm Hg, respectively). IHT has attracted increasing attention as a new exercise method for improving hypertension. In recent years, several meta-analyses have shown that IHT treatment can lead to a sustained decrease in blood pressure (58–60). RT’s reduction of blood pressure may be due to the increased number of metabolites (vasodilators) in skeletal muscle during and after exercise, such as H^+^, ADP, lactate, CO_2_, etc., which contribute to the reduction of blood pressure (61). RT can also regulate the changes of systemic blood circulation through autonomic reflex (such as pressure reflex, metabolic reflex, mechanical reflex, chemical reflex, etc.). During resistance exercise, blood pressure increases, vagal-mediated responses are activated, cardiac variability and contractile activity are reduced, peripheral vascular resistance is lowered, inducing systemic vasodilation and thus lowering blood pressure, and this mechanism is continuously activated as resistance exercise progresses, thus helping to keep blood pressure low after exercise (62–64). Previous studies have shown that HIIT can improve the health parameters of patients with cardiovascular disease better than moderate-intensity AE (65, 66). A recent meta-analysis showed that HIIT can promote a greater reduction in blood pressure in healthy adults or hypertensive patients than moderate-intensity continuous exercise (MICE) (67). Our results show that HIIT is more effective than AE in reducing blood pressure in hypertensive patients (SBP and DBP were –4.23 and –4.57 mm Hg, respectively). In contrast to MICE, HIIT results in an increased blood flow to muscle that promotes an increase in endothelial cell shear stress (mechanical stimulation), which promotes the release of vasodilators such as histamine, and the continuous vasodilator response reduces systemic vascular resistance and blood pressure (68).

### Vascular endothelial function

Vascular endothelium is an active and dynamic tissue that can maintain blood circulation, regulate vascular tone, microvascular permeability, signal transduction, angiogenesis, and inflammatory response (69). Vascular endothelial control of vascular tension is regulated by the production and release of mediators such as nitric oxide, prostacyclin, prostaglandin, thromboxane, angiotensin II, endothelin-1, and reactive oxygen species (70). Compared with healthy people, the number of endothelial progenitor cells (EPCs) is reduced in hypertensive patients, which increases the risk of vascular endothelial dysfunction and atherosclerosis in hypertensive patients (71, 72). FMD is an important non-invasive method for measuring vascular endothelial function (73). Regular exercise can improve vascular endothelial homeostasis, mainly by increasing blood flow and shear stress, reducing reactive oxygen species production, and increasing NO availability in endothelial cells (74). Studies have shown that AE can enhance endothelial function by increasing the lateral shear stress of vascular and upregulating endothelial nitric oxide synthase (eNOS) and NO, thus improving FMD (75). Our study shows that AE increases FMD in hypertensive patients. Of the studies included in this paper, Swift’s study included only menopausal women performing three different amounts of AE (4 kal/kg/week: *MD* = 1.57, 8 kal/kg/week: *MD* = 2.10, 12 kal/kg/week: *MD* = 1.71) and showed that moderate intensity AE better improved vascular endothelial function in hypertensive patients and that the effect of exercise was similar to the total MD (1.56) (40). In addition, regression and subgroup analyses showed that the positive effect of exercise training on FMD was independent of antihypertensive drug use and that the magnitude of FMD elevation in patients not using drugs was similar to the effect of using drugs. Some studies have suggested that exercise increases FMD because exercise increases blood flow and shear force, enhances the activity of eNOS, and thus increases the formation and bioavailability of NO, thus improving endothelial function (76). Whether RT can improve FMD is still debated. For sedentary people, high-intensity RT can reduce FMD and increase arterial stiffness, whereas moderate-intensity RT can improve endothelial function (33, 34). However, our study demonstrates that RT improves FMD in patients with hypertension. During RT, muscle contractions produces resistance resulting in compression blood vessels and transient ischemia, when muscles relax, the release of blood flow produces congestion and increases flow shear stress (77). Therefore, despite their differences, both AE and RT can improve vascular endothelial function. Studies have shown that HIIT can improve the health indicators of patients with cardiovascular disease better than AE (65, 66). Our study shows that HIIT is more effective than AE in improving FMD in hypertension patients (1.92 for AE and 4.31 for HIIT). The mechanism by which HIIT improves endothelial function in hypertensive patients is not completely clear. Recent studies have shown that HIIT can reduce catecholamine levels and α-adrenergic receptor density and increase NO production and NO bioavailability (36, 78).

### Arterial stiffness

PWV refers to the conduction velocity of the pressure wave propagating along the wall of the aorta with each pulse ejection, and it is a non-invasive index for evaluating the stiffness of arterial vessels. PWV can reflect the elastic state of the aorta system and the middle aorta system (79). Arterial stiffness is determined mainly by three factors: structural elements in the arterial wall, such as elastin and collagen, dilatation pressure, and vascular smooth muscle tension (80). The main physiological features of hypertension include increased hardening of the arteries and decreased vascular compliance. Evidence from animal and human studies suggests that exercise has beneficial effects on vascular compliance and remodeling (81). The mechanism of exercise in improving arterial stiffness is complex. Existing studies have shown that regular exercise increases blood flow and thus imposes higher shear stress on endothelial cells, which in turn leads to increased phosphorylation of eNOS and the production of NO, which has beneficial effects on arteriosclerosis through a series of signal transductions (82). Although many RCTs have demonstrated that all types of exercise can improve vascular endothelial function (83–85), the effects of different exercise patterns on arterial stiffness are debated (86–88). Studies have shown that 12 weeks of AE training at 70–75% HRmax can decrease arterial elasticity in healthy adults (89). Other studies have shown that 12 weeks of AE training at 65–70% HRmax does not improve arterial stiffness s in hypertension patients (90). High-intensity RT can reduce FMD and increase arterial stiffness in sedentary people (34). The results of this study show that exercise is effective in improving FMD in hypertensive patients and does not improve the paradoxical phenomenon of PWV. Few studies have examined why exercise can improve FMD without improving or even aggravating arterial stiffness. The reasons why exercise improves FMD but does not improve or even exacerbate PWV are less well studied, with Soltész et al. (91). reporting a negative correlation between FMD and PWV and Koivistoinen et al. (92) finding no direct correlation between FMD and PWV. Some studies have shown that vascular endothelial function is mediated by rapid changes in cell signals under exercise intervention (93) whereas changes in stiffening of the arteries usually involve remodeling of the extracellular matrix of the arterial wall, which usually takes a long time. For example, AE or other healthy-lifestyle interventions usually take 3 months or more to decrease arterial stiffness (94). Our study concluded that FMD measurements are dependent on changes in vascular endothelial contraction and diastole, and that arterial stiffness is influenced by multiple structural changes in connective tissue proteins within the arterial wall mesothelium, endothelium, and smooth muscle cells (95). Thus, although exercise is effective in improving FMD in hypertensive patients, the impact of improved endothelial function is limited due to hypertension-mediated mechanical stress leading to elastin disruption, collagen deposition, and fibrosis, which leads to progressive arterial stiffness (96), and this chronic sclerosis may take longer to improve or even be irreversible.

### Limitations

Although this paper comprehensively explores the effect of different modalities of exercise on the improvement of vascular function in pre- and hypertensive patients. However, there are still shortcomings, and the limitations may have affected this study’s conclusions and implications. First, in order to fully reveal the different effects of different exercise modalities on vascular function in pre- and hypertensive patients, this study considered a direct comparison of the effects of different exercise regimens using a net meta, but due to the small amount of included studies, a net meta-analysis could not be performed. Second, the reviewed literature exhibited differences in study design, such as exercise mode and intensity, frequency, and duration of intervention, which may have resulted in this study’s heterogeneity. Finally, because few studies have examined combined exercise, no definitive results concerning the effect of combined exercise on vascular function in hypertensive patients could be obtained.

## Conclusion and suggestions for future research

The results of this study showed that AE, RT, and HIIT all improved SBP, DBP, and FMD in hypertensive patients, but not PWV, and that this effect was independent of the subjects’ medication status, baseline SBP, age and duration of exercise. Although this study found that combined exercise also reduce SBP, DBP, and improved FMD in hypertensive patients, it was not possible to draw strict conclusions about whether combined exercise improved vascular function due to the lack of studies on combined exercise. In order to fully explain the paradoxical phenomenon that exercise enhances FMD without reducing PWV in pre- and hypertensive patients, more research is needed to investigate in depth which exercise modality can improve hypertension-mediated vascular dysfunction and vascular sclerosis, and to provide more scientific and effective exercise programs for hypertensive patients.

## Author contributions

HZ and SW: collection, sorting, and analysis of raw materials. HH: thesis design and revision. CZ: critical revision of manuscripts with important knowledge contents. All authors final draft approval.
