# Supplementary material for: Effect of exercise on vascular function in hypertension patients: A meta-analysis of randomized controlled trials
Source: Front Cardiovasc Med. 2022 Dec 21;9:1013490. doi: 10.3389/fcvm.2022.1013490 (PMC9812646; doi:10.3389/fcvm.2022.1013490)
Supplement: Supplementary file 1 [file Data_Sheet_1.docx]

Supplementary Material

## Supplementary Figures


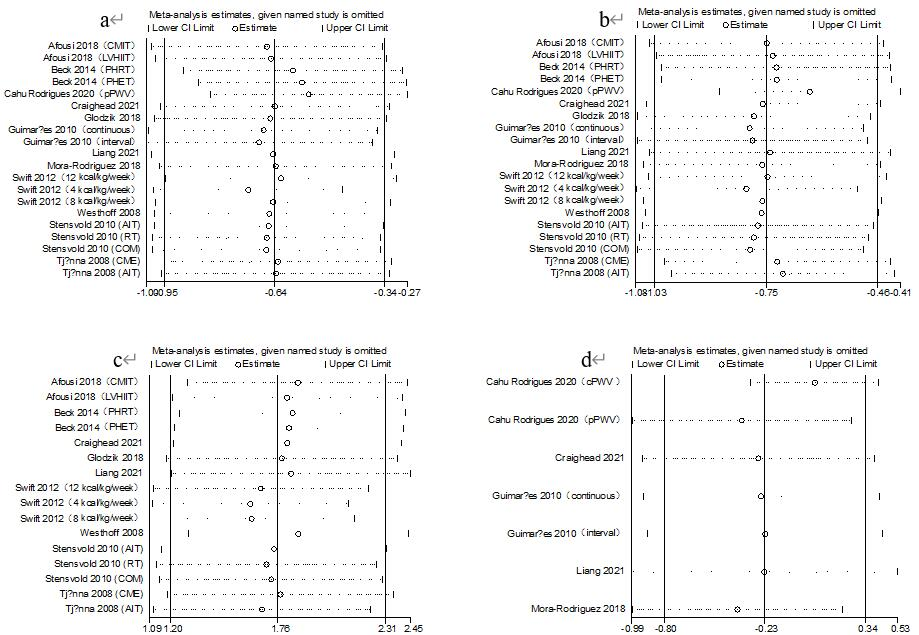


**Supplementary Figure 1.** Sensitivity analysis of the effect of exercise on vascular function in hypertensive patients. (a) SBP; (b) DBP; (c) FMD; (d) PWV.


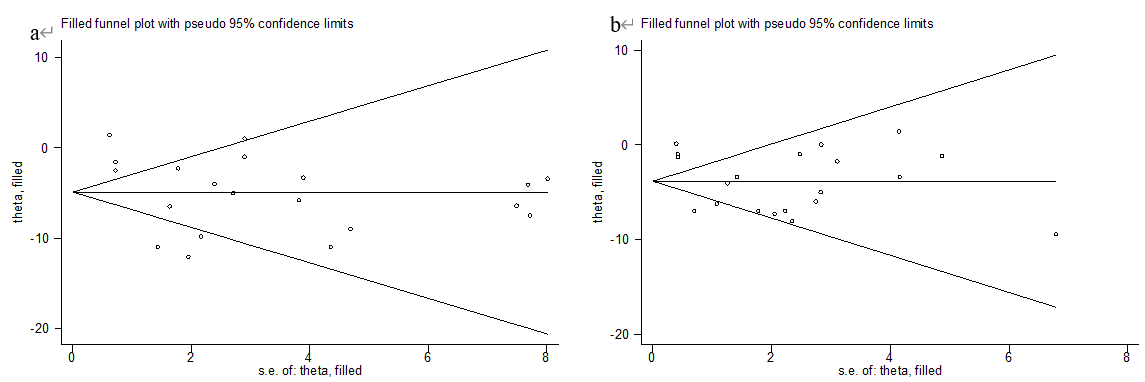


Figure S2. Funnel diagram of the effect of exercise on vascular function in hypertensive patients. (a) SBP; (b) DBP.
